# Effects of Breeds on the Content of Functional Nutrition in Eggs

**DOI:** 10.3390/ani13193066

**Published:** 2023-09-29

**Authors:** Caiyun Jiang, Ruochen Chen, Xuefeng Shi, Longyu Zhuang, Chen Zhou, Wenbin Zhou, Junying Li, Guiyun Xu, Jiangxia Zheng

**Affiliations:** College of Animal Science and Technology, China Agricultural University, Beijing 100193, China; cyjiang@cau.edu.cn (C.J.); tianxietianyu@163.com (R.C.); xuefeng.shi@cau.edu.cn (X.S.); zhuanglongyu@126.com (L.Z.); chendi259858158@163.com (C.Z.); 2020304010114@cau.edu.cn (W.Z.); lijunying@cau.edu.cn (J.L.); ncppt@cau.edu.cn (G.X.)

**Keywords:** functional nutrition eggs, different breeds, n-3 PUFA, selenium, lutein

## Abstract

**Simple Summary:**

n-3 PUFA, selenium and lutein cannot be synthesized in the human body, but these substances are very important for human health. Consuming functional eggs enriched with nutrients can increase the body’s intake of these substances. To efficiently produce functional eggs, selecting breeds that can deposit higher levels of these nutrients is necessary. Therefore, we investigated the types, dosages, and cycles of additives used in the production of functional eggs. In Trial 1, we investigated the differences in n-3 PUFA deposition among five breeds of eggs. The results showed significant variations in the ability of different breeds to deposit n-3 PUFA in eggs, with Dwarf Layer demonstrating a significant advantage. In Trial 2, we investigated the difference of n-3 PUFA, selenium and lutein deposition in eggs from two reeds in terms of their ability to deposit selenium and lutein in eggs. Additionally, the n-3 PUFA content in eggs from the two breeds was similar to the findings in Trial 1. Therefore, this experiment provides valuable insights for selecting different breeds in the production of functional eggs, ultimately improving the quality and economic benefits of the production process.

**Abstract:**

The purpose of this study was to compare the differences in the content of functional nutrients in eggs, performance parameters, and egg quality parameters of different chicken breeds. In Trial 1, 150 41-week-old hens of each breed, including the Dwarf Layer, White Leghorn, Silky fowl, Beijing-you chicken, and Shouguang chicken, were randomly assigned to the control (CON) and 2.5% flaxseed oil (FSO) groups to compare the difference in n-3 polyunsaturated fatty acid (PUFA) content in eggs. The contents of α-linolenic acid (ALA), eicosapentaenoic acid (EPA), docosahexaenoic acid (DHA), and total n-3 PUFA in eggs were increased (*p* < 0.05) in the FSO groups. The ALA (4.28%), DHA (2.03%), and total n-3 PUFA (6.46%) contents in eggs of Dwarf Layer were the highest among the five breeds (*p* < 0.05). To further verify if other functional nutrients also have such differences, 600 24-week-old White Leghorn and Dwarf Layer were allocated to four groups: CON, FSO, 0.02% selenium-enriched yeast (SEY), and 0.20% marigold flower extract (MFE), in Trial 2. The content of functional nutrients in eggs was significantly increased (*p* < 0.05) after feeding these additions. After feeding FSO, the eggs of the Dwarf Layer had a higher n-3 PUFA content than the White Leghorn (*p* < 0.05). However, no significant differences were found in selenium and lutein content in different breeds. Performance and egg quality were not negatively impacted by FSO, SEY, or MFE.

## 1. Introduction

Eggs enriched with one or more functional ingredients, namely, n-3 polyunsaturated fatty acid (PUFA), eicosapentaenoic acid (EPA), docosahexaenoic acid (DHA), selenium, and lutein, have enhanced nutritional value within the context of human health [1]. In recent years, functional nutrition of eggs has garnered the attention of many researchers [1,2,3,4,5,6,7,8,9]. n-3 PUFA reduces the levels of plasma lipids and improves cardiovascular health, thereby reducing disease severity [10] and serum concentrations of alanine aminotransferase and improving liver function in patients with non-alcoholic fatty liver disease [11].

The consumption of eggs rich in n-3 PUFA is an effective way to acquire this functional ingredient [12]. The most widely used method to produce eggs enriched with n-3 PUFA is to include sources of n-3 PUFA in the diet of hens [13]. Among the different dietary sources of n-3 PUFA and α-linolenic acid (ALA) are present in plant-based oilseeds, whereas EPA and DHA are found primarily in marine oils and algae. In addition, EPA and DHA are also found in plant oils derived from certain genetically modified organisms, such as canola [14] and arabidopsis seeds [15]. However, the utilization of such oils containing EPA and DHA has not yet been commercialized. In one study, when the same amount of extra n-3 PUFA (120 mg per 100 g feed) was added to the diet, the deposition of n-3 PUFA was the highest in fish oil, followed by microalgae, and lowest in flaxseed [16]. By adding the same amount of fish oil or flaxseed oil (FSO) (3%), more n-3 PUFA can be deposited in the egg yolk with FSO (7.60%) than with fish oil (4.17%), and the eggs from hens given feed with 3% fish oil were unacceptable to sensory panelists [17]. Flaxseed is one of the most important oilseed crops, a rich source of ALA and is emerging as an important functional food ingredient [18]. However, flaxseed contains linatine (a vita-min B6 antagonist) as well as anti-nutritional factors, including insoluble and soluble non-starch polysaccharides [19]. Non-starch polysaccharides in flaxseeds decrease nutrient digestibility and can negatively impact egg n-3 PUFA incorporation [20]. However, it was reported in some studies that the results obtained were differentiated due to some unknown reasons yet. A recent study reported that high content of flaxseed combined with another antioxidant source (sea buckthorn or grapeseed) had positive effects on production performances, fatty acids deposition, and health indexes [21]. Research has shown that dietary FSO can be a viable option for enriching eggs with ALA. This is because FSO contains a higher amount of ALA than milled flaxseed, resulting in increased ALA deposition in yolks [3,18]. Furthermore, incorporating dietary FSO in the feed of laying hens can be an effective means of promoting the enrichment of n-3 PUFA in egg yolks [22].

Selenium is known for its antioxidative properties, as it protects organisms from the harmful effects of free radicals and carcinogens [23]. To improve human selenium status, foods such as meat, milk, and eggs that have been enriched with selenium can be consumed [23]. In the poultry industry, it is common to supplement the diets of laying hens with selenium to enhance the selenium concentration in eggs and meat. Traditionally, sodium selenite has been the go-to source of selenium for animal feeds. However, in recent times, organic sources of selenium, such as selenium-enriched yeast (SEY), have gained traction as an approved means of increasing the selenium concentration in eggs and carcass meat [6,7]. As such, SEY supplementation can be implemented as part of selenium-enriched egg production [7,8].

Lutein has been shown to possess anti-inflammatory properties, making it useful in the treatment of a variety of inflammatory disorders, such as diabetes retinopathy, eye diseases, liver injury, and obesity [24,25]. Given that the synthesis of lutein within the human body is not possible, the only means of acquiring it is through the consumption of dark green leafy vegetables and egg yolks [26,27,28]. Marigold flowers are an excellent natural source of lutein, and they are often utilized to enhance the color of the yolk and carotenoid content of eggs [4,29].

According to observations, it has been found that only a few studies have compared these egg-related indices across different breeds (i.e., within the context of dietary supplementation). It is important to compare the differences in the content of functional nutrients in enriched eggs from different breeds to produce eggs with enhanced nutritional profiles. Dwarf Layers are known for their good egg-laying performance, and their eggs have a pink shell color, which is preferred by Chinese consumers [30]. Silky fowl, Beijing-you chicken and Shouguang chicken are local chicken breeds in China. Although they may not have high egg-laying performance, their eggs are priced higher. Considering the market potential for functional nutrition eggs, these breeds can be considered candidate chicken breeds. White Leghorn is a commonly used breed for functional egg research [31,32,33]. Therefore, we have selected the above five breeds to explore the differences in the ability of different breeds to deposit functional nutrients. The aim is to provide some insights and references for the production of functional nutrition eggs. 

## 2. Materials and Methods

### 2.1. Animal Ethics

The animal experiments in this study adhered to the Guidelines for Experimental Animals provided by the Animal Care and Use Committee of China Agricultural University, with permit number AW10803202-1-2. The experiments also complied with the Animal Research: Reporting of In Vivo Experiments (ARRIVE) guidelines.

### 2.2. Experimental Materials and Feeding Management

The animal experiment took place at the Experimental Unit for Poultry Genetic Resource and Breeding, a facility belonging to China Agricultural University. The population used in the experiment was produced via pedigree mating, with each generation being the result of a random breeding process. The Dwarf Layer was developed by the Poultry Genetic Resources and Breeding Experimental Unit of China Agricultural University through 4 repeated backcrosses of the meat type dwarf ISA-Vedette to the female CAU brown egg layer, mainly used for egg production [34,35]. Silky fowl, Beijing-you chicken, and Shouguang chicken are all local chicken breeds in China. White Leghorn is a traditional and commonly used white shell layer. All chicken breeds used in the experiment are pure lines, among which only Dwarf Layers have dwarf genes.

In Trial 1, 150 laying hens (41 weeks old) of each breed were assigned at random to the FSO group, which was fed the basic diet mixed with 2.5% flaxseed oil and 0.016% vitamin E, and the CON group, which was fed the basic diet (n = 75 in each group). The breeds used were Dwarf Layer, White Leghorn, Silky Fowl, Beijing-you chicken, and Shouguang chicken. Flaxseed oil was obtained from Ningxia Qianhoufu Trading Co., Ltd. (Yinchuan, Ningxia, China). To avoid a negative change in appearance, a 2.5% inclusion level was implemented in this study based on findings from a prior publication, where 3% flaxseed oil inclusion resulted in an adverse effect on appearance [17]. The addition of vitamin E is a traditional way to relieve PUFA oxidation and improve egg storage quality and oxidation stability [36]. After 28 days of feeding, 30 eggs per breed and group were collected at random to confirm n-3 PUFA enrichment and egg equality. Weekly evaluations were carried out to measure their feed consumption, egg laying rate and egg weight for each group (n = 75 in each group). These measurements were recorded throughout the entire 28-day experimental period.

In Trial 2, 300 White Leghorn (24-week-old) and 300 Dwarf Layer (24-week-old) chickens were distributed within each breed into four groups: (1) CON; (2) FSO; (3) SEY, fed basic diet mixed with 0.02% selenium-enriched yeast (2000 mg/kg); 4) MFE, fed basic diet mixed with 0.2% marigold flower extract (1.5%). Flaxseed oil was obtained from Ningxia Qianhoufu Trading Co., Ltd. (Yinchuan, Ningxia, China). SEY was obtained from Yunnan Dongcheng Lvkang Biotechnology Co., Ltd. (Zhaotong, Yunnan, China). MFE was obtained from Hebei Meineier Biotechnology Co., Ltd. (Shijiazhuang, Hebei, China). After 28 days of feeding, 30 eggs per breed and group were collected at random to confirm n-3 PUFA enrichment and egg equality. Weekly evaluations were carried out to measure their feed consumption, egg laying rate and egg weight for each group (n = 75 in each group). These measurements were recorded throughout the entire 28-day experimental period.

The basic diet used in this study was formulated to meet the nutritional standards recommended by the National Research Council (1994) and the feeding standards for chickens (NY/T 33-2004). Please refer to Table 1 for details on the composition and nutritional content of the basic diets. Metabolizable energy is calculated based on the Chinese feed composition and nutritional value table. The CON and FSO groups are illustrated in Table 2, which displays the percentage composition of fatty acids. Throughout the 28-day study period, the chickens were kept individually in cages, with one chicken per cage. The recommended feeding schedule is twice a day (08:30, 14:30), and the eggs should be collected once a day (16:30). A nipple-type water dispenser is used to provide free drinking water for the hens. The light system consists of a morning and evening light supplement system, with a light duration of 16 h per day and a light intensity of 10 Lx. It is important to check the hens daily to ensure they have sufficient feed and water supply, as well as to monitor their health status. 

### 2.3. Sample Collection

A plastic hand-held egg separator (Wuxi, Jiangsu, China) was applied to separate various egg components, such as yolk and albumen. In Trial 1, after the albumen of all groups was eliminated, the yolks were weighed and stored at −20 °C until subsequent analyses were performed. The albumen that had adhered to the eggshell was wiped with a paper towel, and then the eggshell was weighed after 12 h. For Trial 2, the albumen from the FSO, MFE, and a portion of the CON groups were removed, while the yolks were weighed and stored at −20 °C until further analysis. The egg yolks and albumen from another part of the CON group were mixed thoroughly and then stored at −20 °C for subsequent analysis. The egg yolks and albumen from the SEY group were mixed thoroughly and then stored at −20 °C for subsequent analysis. 

### 2.4. Egg Quality Determination

The evaluation of egg quality involved collecting 30 eggs from each dietary group, which were then analyzed for various quality parameters such as eggshell thickness (EST), eggshell strength (ESS), yolk color (YC), egg weight (EW), egg yolk weight (EYW), albumen weight (AW), albumen height (AH), and Haugh units (HU). The EW, AH, HU, and YC measurements were obtained using a multifunctional egg tester (EMT-5200; Tokyo, Japan), while ESS was determined using a Model-II eggshell strength tester (Robotmation, Tokyo, Japan). EST was measured at three different positions on the egg (Blunt end, equatorial, sharp end) using a micrometer (Robotmation, Kyoto, Japan), and the average value was calculated for further analysis.

### 2.5. Nutrient Content Determination

Ten eggs from each group were used to determine the n-3 PUFA, lutein, and selenium concentrations in the egg. The separated egg yolks from CON and FSO groups were freeze-dried at −80 °C for 72 h with a vacuum freeze-dryer and weighed. Then, the lyophilized egg yolks were crushed into powder, and fatty acids were determined through gas chromatography (Agilent 6890, Agilent Technologies Inc., Santa Clara, CA, USA), following the national standard GB-5009.168-2016. In brief, 0.5 g of the sample was accurately weighed into a screw-top glass tube, and then toluene and acetyl chloride methanol solution (10%) were added. After mixing, the sample was allowed to stand in an 80 °C water bath for 2 h. Next, the reaction solution was transferred to a centrifuge tube, and the glass tube was washed with sodium carbonate solution. Finally, 100 μL of the upper clear liquid was taken, filtered through a membrane, and analyzed using a gas chromatograph to determine the content of fatty acids.

The samples from the CON and SEY groups were removed, and the selenium content was determined using a fluorescence spectrophotometer (AF 7500, Beijing Titan Instruments Co., Ltd., Beijing, China), following the national standard GB-5009.93-2017. The experiment involved digesting 1 g of the sample with a mixture of nitric and perchloric acids (*v*/*v* = 9:1), followed by adding hydrochloric acid and ethylene diamine tetraacetic acid solution, and then adding 2,3-diaminonaphthalene reagent. Following purification, the sample was subjected to measurement of fluorescence intensity, with an excitation wavelength of 376 nm and an emission wavelength of 520 nm.

The separated egg yolk from the CON and MFE groups was removed, and lutein content was determined through high-performance liquid chromatography (HPLC 1260, Agilent Technologies, Inc., Santa Clara, CA, USA), following the national standard GB-5009.248-2016. In summary, homogenized egg samples weighing 2 g were mixed with 0.2 g of butylated hydroxytoluene and 10 mL of anhydrous ethanol in a 50 mL polypropylene centrifuge tube. Then, 10 mL of 10% potassium hydroxide solution was added for the saponification reaction. After the reaction, the extract was obtained using a solvent extraction method. Then, after washing and concentration treatment, the extract was used for liquid chromatography analysis with 0.1% butylated hydroxytoluene ethanol solution as the base solution. For separation, a column (4.6 × 250 mm, 5 μm) was utilized with methanol/water and methyl tert-butyl ether mixture as the mobile phase, and the detection wavelength was set at 445 nm.

The contents of three functional nutrition ingredients in the egg were calculated as follows:

Fatty acid content (%) = [fatty acid content in the sample (mg/g)]/[total fatty acid content in the sample (mg/g)].

Selenium content (μg/egg) = the selenium content in the sample (μg/g) × egg weight (g). 

Lutein content (μg/egg) = the lutein content in the sample (μg/egg) × egg yolk weight (g).

### 2.6. Statistical Analyses

All test data were all analyzed using the univariant program of the general linear model process of SPSS17.0, with two-way ANOVA for diets and chicken breeds. The Duncan multiplex test was performed to identify indicators with significant main effects. A predetermined level of statistical significance was set at *p* < 0.05. GraphPad Prism 8 was used as the analytical tool for data plot analysis.

## 3. Results

### 3.1. Effect of FSO on Performance Parameters and Egg Quality Parameters 

The impact of FSO feeding on performance and egg quality parameters in Trial 1 is illustrated in Table 3 and Table 4, while Table 5 and Table 6 depict the effects observed in Trial 2. Feeding FSO resulted in a significant increase in average daily feed intake in White Leghorn (88.01 g vs. 111.79 g), egg mass of White Leghorn (29.98 g/d/bird vs. 37.94 g/d/bird) and Silky fowl (19.61 g/d/bird vs. 23.04 g/d/bird), egg production of White Leghorn (52.98% vs. 67.12%) and Silky fowl (44.25% vs. 52.83%) were observed. Furthermore, there was a significant decrease in the feed-to-egg ratio of Silky fowl (4.60 vs. 3.81) and the average daily feed intake of the Dwarf Layer (110.67 g vs. 104.06 g). Furthermore, there was a significant increase in EST of White Leghorn (330.39 μm vs. 345.77 μm), EYW of Dwarf Layer (16.10 g vs. 16.76 g), EW (48.59 g vs. 50.43 g) and AW (27.94 g vs. 29.29 g) of Shouguang chicken. Conversely, a significant decrease in EST was observed in the Dwarf Layer (323.38 μm vs. 310.31 μm). The results of Trial 1 demonstrated that FSO did not significantly affect ESS (*p* > 0.05), YC (*p* > 0.05), AH (*p* > 0.05), and HU (*p* > 0.05) across all five breeds. Feeding the same diet, the highest average daily feed intake was observed in Shouguang chicken, the highest number of EST was observed in Beijing-you chicken, and the higher number of ESS was exhibited in three local Chinese breeds. A higher number of YC was seen in the Beijing-you and Shouguang chickens. The highest number of EW and EYW were observed in White Leghorn. A higher number of AW was found in the Dwarf Layer and White Leghorn. Additionally, the highest number of AH and HU were exhibited in Dwarf Layer. In the CON group, the highest feed-to-egg ratio was displayed in Silky fowl, and the highest number of egg mass and egg production were in the Dwarf Layers. In the FSO group, the highest feed-to-egg ratio and egg production were observed in Shouguang chickens, and the highest egg mass was in White Leghorns.

On the other hand, it did not exhibit any statistically significant differences in the average daily feed intake, egg mass, feed-to-egg ratio, egg production, or egg quality parameters (*p* > 0.05) after feeding FSO in Trial 2. No significant differences were observed in performance, YC, and EYW between the two breeds fed the same diet. In the CON group, the highest number of AW, AH, and HU were exhibited in Dwarf Layer. In the FSO groups, the highest number of ESS was shown in White Leghorn, while the highest number of AH and HU were shown in Dwarf Layer. The main distinction between the two breeds was primarily concentrated in egg quality.

### 3.2. Dwarf Layer Can Deposit More n-3 PUFA in Eggs Than Other Breeds

**Figure 1:**
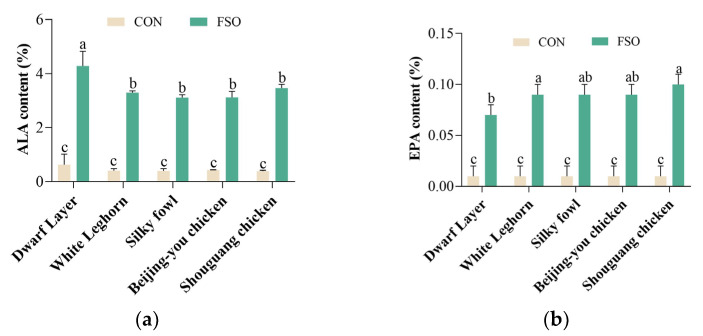

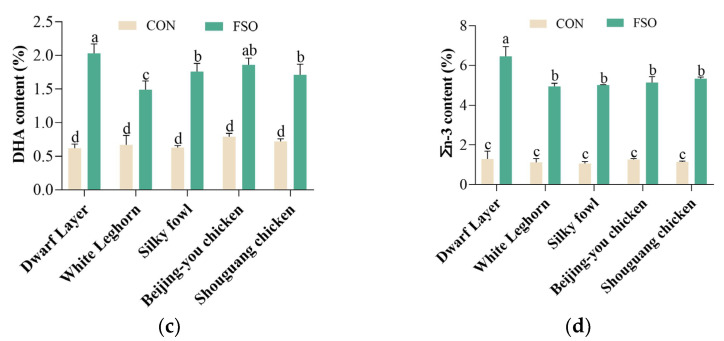
Egg fatty acids composition (%) of different breeds with FSO dietary in Trial 2. (**a**) ALA content (%) of five breeds with FSO dietary; (**b**) EPA content (%) of five breeds with FSO dietary; (**c**) DHA content (%) of five breeds with FSO dietary; (**d**) ∑n-3 content (%) of five breeds with FSO dietary. Results are shown as mean ± SD, n = 15; “a, b, c, d” means with different superscripts indicate significant differences (*p* < 0.05). ALA = α-linolenic acid (C18:3); EPA = eicosapentaenoic acid (C20:5); DHA = docosahexaenoic acid (C22:6); ∑n-3 = ALA + EPA + DHA; CON = basic diet; FSO = basic diet + 2.5% flaxseed oil + 0.016% Vitamin E.

The results of fatty acid composition (%) of eggs from different breeds fed the FSO diet in Trial 1 are presented in Figure 1 and Table S1, and the fatty acid composition of eggs (mg/egg; mg/g yolk; mg/g egg; mg/100 g egg) in Trial 1 is shown in Table S2. The percentage of ALA, EPA, DHA, and total n-3 PUFA in eggs was not significantly different between the five breeds when fed a basic diet. After the hens were fed with FSO, the distribution of various fatty acids in eggs changed significantly; the range of saturated Fatty Acid (SFA) decreased (from 36.46–37.92% to 32.96–35.50%), and that of PUFA increased (from 19.90–22.35% to 23.31–26.20%) increased (Table S1). PUFA in eggs was mainly represented by n-3 and n-6 PUFA. After feeding hens with FSO, the percentage of n-3 PUFA in eggs significantly increased (*p* < 0.05), and the n-6 PUFA-to-n-3 PUFA ratio significantly decreased (from 16.36–19.99% to 3.02–4.09%) (*p* < 0.05) (Table S1). The total n-3 PUFA percentage in egg significantly increased for all breeds, from 1.29% to 6.46% for Dwarf Layer (*p* < 0.05); 1.12% to 4.94% for White Leghorn (*p* < 0.05); 1.06% to 5.02% for Silky fowl (*p* < 0.05); 1.26% to 5.14% for Beijing-you chicken (*p* < 0.05); and 1.15% to 5.33% for Shouguang chicken (*p* < 0.05) (Figure 1). Moreover, the increase was the biggest in the Dwarf Layer.

The contents of n-3 PUFA components (i.e., ALA, EPA, and DHA) in eggs without FSO were 0.39–0.63%, 0.01%, and 0.62–0.79%, respectively. After feeding FSO, the ALA, EPA, and DHA contents were 3.11–4.28%, 0.07–0.10%, and 1.49–2.03%, respectively (Figure 1). After feeding hens with FSO, the percentage of ALA, EPA, DHA, and total n-3 PUFA in eggs increased significantly (*p* < 0.05) and showed a significant difference between breeds (*p* < 0.05). The percentage of ALA (4.28%), DHA (2.03%), and total n-3 PUFA (6.46%) in the eggs of Dwarf Layer were the highest among those of the five breeds (*p* < 0.05). For the percentage of EPA in eggs, the order from high to low was: Shouguang chicken (0.10%), White Leghorn (0.09%), Silky fowl (0.09%), Beijing-you chicken (0.09%), and Dwarf Layer (0.07%) (Figure 1b). A comparison of the other four units showed that the egg of the Dwarf Layer contains more n-3 PUFA than the other four breeds after feeding FSO (Table S2). 

**Figure 2:**
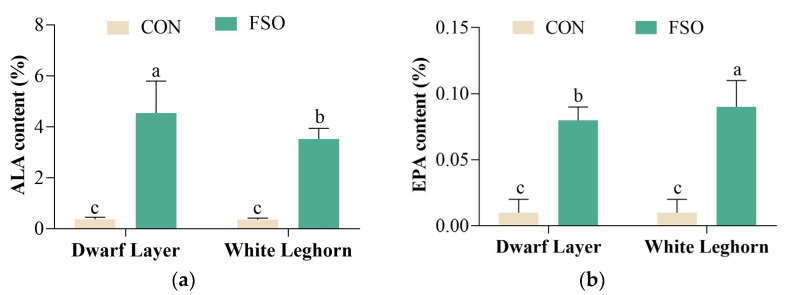

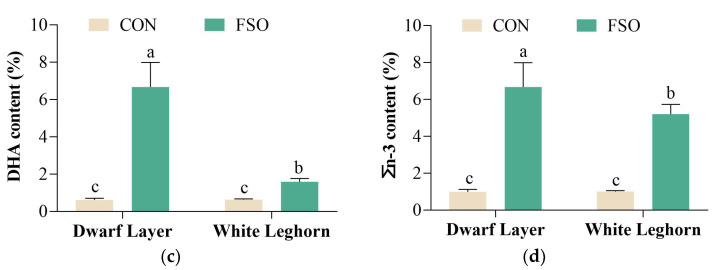
Egg fatty acids composition (%) of different breeds with FSO dietary in Trial 2. (**a**) ALA content (%) of two breeds with FSO dietary; (**b**) EPA content (%) of two breeds with FSO dietary; (**c**) DHA content (%) of two breeds with FSO dietary; (**d**) ∑n-3 content (%) of two breeds with FSO dietary. The results are presented as mean ± SD, n = 10; “a, b, c” means with different superscripts indicate significant differences (*p* < 0.05). ALA = α-linolenic acid (C18:3); EPA = eicosapentaenoic acid (C20:5); DHA = docosahexaenoic acid (C22:6); ∑n-3 = ALA + EPA + DHA; CON = basic diet; FSO = basic diet + 2.5% flaxseed oil + 0.016% Vitamin E.

The effect of feeding FSO of two different breeds on egg fatty acids (%) in Trial 2 was shown in Figure 2 and Table S3, and the fatty acid composition of eggs (mg/egg; mg/g yolk; mg/g egg; mg/100 g egg) was shown in Table S4. Similar to Trial 1, the egg of the Dwarf Layer contains more n-3 PUFA than White Leghorn after feeding FSO.

### 3.3. Effect of SEY on Performance Parameters and Egg Quality Parameters

The effects of feeding SEY on laying performance and egg quality are summarized in Table 5 and Table 6. After feeding SEY, the egg mass of the Dwarf Layer (39.93 g/d/bird vs. 44.63 g/d/bird), the egg production of the Dwarf Layer (79.55% vs. 88.44%), the average daily feed intake of White Leghorn (108.51 g vs. 116.13 g) increased significantly (*p* < 0.05). There was no significant effect in feed-to-egg ratio, ESS, YC, AW, AH, and HU of any breeds by feeding SEY. However, a significant increase in EYW was observed in the Dwarf Layer (13.19 g vs. 14.11 g). There were no significant differences in performance, ESS, and YC between the two breeds fed the same diet. However, in the CON group, the highest number of AW (31.42 g vs. 29.63 g), AH (7.11 mm vs. 6.67 mm), and HU (87.97 vs. 85.20) was exhibited in the Dwarf Layer. In the SEY groups, the highest number of EW (51.71 g vs. 48.10 g), EYW (14.11 g vs. 13.04 g), AW (32.38 g vs. 29.77 g), AH (7.57 mm vs. 6.54 mm), and HU (89.118 vs. 84.38) were showed in Dwarf Layer.

### 3.4. Selenium Deposition in Different Breeds

**Figure 3:**
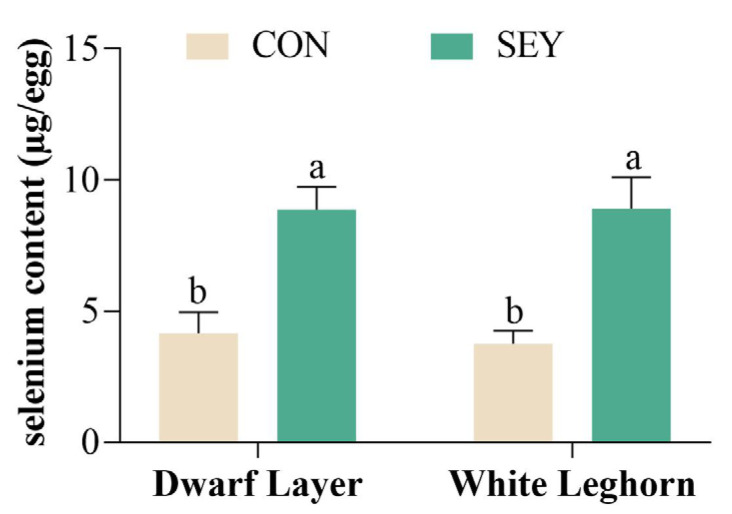
Selenium deposition in eggs between breeds. Results are shown as mean ± SD, n = 10; “a, b” means with different superscripts indicate significant differences (*p* < 0.05). CON = basic diet; SEY = basic diet + 0.02% selenium yeast.

The effects of feeding SEY among different breeds on selenium deposition in eggs are shown in Figure 3. After feeding SEY, the content of selenium in eggs significantly increased from 4.16 μg/egg to 8.87 μg/egg for the Dwarf Layer (*p* < 0.05) and 3.76 μg/egg to 8.90 μg/egg for White Leghorn (*p* < 0.05). However, the selenium content in the eggs was not significantly different between breeds fed CON and SEY (*p* > 0.05).

### 3.5. Effect of MFE on Performance Parameters and Egg Quality Parameters

The effects of feeding MFE on performance and egg quality parameters are presented in Table 5 and Table 6. The performance parameters (average daily feed intake and feed-to-egg ratio) and egg quality parameters (ESS, EW, EYW, AW, AH, and HU) of the breeds were not affected by feeding MFE. After feeding MFE, the egg mass (CON vs. FSO, 39.93 g/d/bird vs. 44.17 g/d/bird) and egg production (79.55% vs. 87.11%) of Dwarf Layer, the YC of Dwarf Layer (8.26 vs. 9.26) and White Leghorn (8.23 vs. 9.06) increased significantly. There were no significant differences in production performance and YC between the two breeds fed the same diet. In the CON groups, the highest number of AW (31.42 g vs. 29.63 g), AH (7.11 mm vs. 6.67 mm), and HU (87.97 vs. 85.20) were shown in Dwarf Layer. In the MEF groups, the highest number of EW (50.52 g vs. 47.62 g), EYW (13.77 g vs. 13.08 g), AW (31.86 g vs. 29.33 g), AH (7.34 mm vs. 6.42 mm), and HU (88.28 vs. 83.59) were exhibited in Dwarf Layer.

### 3.6. Lutein Deposition in Different Breeds

**Figure 4:**
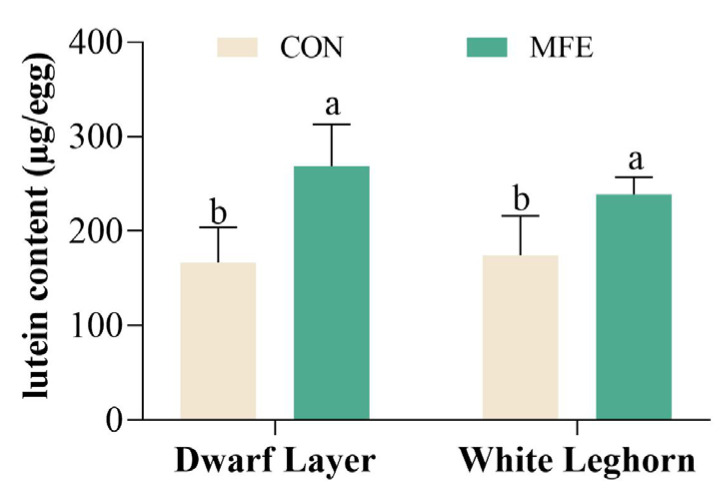
Lutein deposition in eggs between breeds. Results are shown as mean ± SD, n = 10; “a, b” means with different superscripts indicate significant differences (*p* < 0.05). CON = basic diet; MFE = basic diet + 0.2% marigold flower extract.

The effect of feeding MFE among different breeds on lutein deposition in eggs is shown in Figure 4. After feeding MFE, the content of lutein in eggs significantly increased from 166.80 μg/egg to 268.80 μg/egg for Dwarf Layer (*p* < 0.05) and 174.60 μg/egg to 238.70 μg/egg for White Leghorn (*p* < 0.05). However, the lutein content in the eggs was not significantly different between breeds fed CON and MFE diets (*p* > 0.05).

## 4. Discussion

Research has indicated that incorporating n-3 PUFA sources into the diet of laying hens does not have a notable impact on either egg production or egg quality [13,20,37,38]. However, it has been observed that the inclusion of n-3 PUFA sources decreases EST and YC and increases AH and egg production [39]. In Trial 1 of the present study, the effects of dietary FSO on egg production and quality parameters varied depending on the breed of chicken. There were no substantial effects of a diet containing FSO on some measures, such as ESS, YC, and AH. However, there were significant effects on other parameters, including the average daily feed intake of Dwarf Layer and White Leghorn, feed-to-eggs ratio of Silky fowl, egg mass and egg production of White Leghorn and Silky fowl, EST of the Dwarf Layer and White Leghorn, EYW of Dwarf Layer, and AW of Shouguang chicken. In Trial 2, no significant effects of dietary FSO were observed on performance and egg quality parameters in White Leghorn or Dwarf layer breeds. This lack of significant effects may be attributed to differences in chicken breeds, ages, sources of n-3 PUFA, and varying dosages of supplementation used across studies.

In our study, supplementing the diet of hens with FSO led to a significant increase in the ALA, EPA, DHA, and total n-3 PUFA contents in eggs (*p* < 0.05), consistent with previous findings [40,41,42]. However, Lee et al. (2021) reported that in their study, only DHA and n-3 PUFA contents were significantly increased after feeding Hy-Line Brown laying hens with FSO at varying doses (0.2%, 0.4%, 0.6%, and 0.8%) for 4 weeks, while there was no significant difference in the ALA (0.23 vs. 0.20) or EPA (0.01 vs. 0.01) content [22]. This discrepancy may have been due to differences in the doses of FSO added between the studies. Additionally, in the present study, FSO (2.0%) supplementation resulted in the content of SFA in the egg yolk lipids, as was observed by a significant reduction in myristic (0.69 vs. 0.52) and palmitic acid (27.53 vs. 25.70) percentages (*p* < 0.05), consistent with the results of the study by Souza et al.’s research (2008) [43]. In addition, our study demonstrated that the supplementation of FSO in hen diets substantially augmented the n-3 PUFA content in eggs, which corresponded to a significant reduction in the n-6 PUFA-to-n-3 PUFA ratio (*p* < 0.05), akin to the effects of other dietary sources of n-3 PUFA [20,22,31,32,40].

Supplementation of SEY significantly increased the amount of selenium in eggs (*p* < 0.05), which is consistent with other findings [6,7,8,44]. There were no notable impacts observed on ESS, AH, YC, or HU as a result of dietary SEY. This is in agreement with previous reports, which have also observed no significant effects of SEY on these particular parameters [5,7,9]. However, in this study, the EW of the Dwarf Layer breed was significantly altered after supplementation with SEY (*p* < 0.05). When it comes to selenium deposits, there was no significant difference observed in selenium deposits among various chicken breeds (*p* > 0.05). 

The study showed that no significant effects of dietary MFE were observed on ESS, AH, HU, or EW, which aligns with the results of previous research by Grcevic et al. (2019) [4] and Wen et al. (2021) [45]. However, feeding hens with MFE did significantly enhance yolk color, which is in line with earlier studies conducted by Grcevic et al. (2019) (0 vs. 1 g/kg of marigold extract, 9.63 vs. 12.77; 0 vs. 2 g/kg of marigold extract, 9.63 vs. 13.5) [4], Islam et al., (2017) [46], and Wen et al., (2021) [45] (*p* < 0.05). Interestingly, the color of the egg yolk also significantly improved in Dwarf Layer (8.3 vs. 9.3) and White Leghorn (8.2 vs. 9.1). There are certain differences from previous research results, which may be caused by factors such as the source of added lutein and dosage. Feeding with MFE led to a meaningful increase in lutein levels found in eggs (166.8–174.6 μg/egg to 238.7–268.8 μg/egg) (*p* < 0.05), which aligns with the results reported by Wen et al. (2021) [45]. However, lutein deposits did not vary significantly among the chicken breeds (Dwarf Layer vs. White Leghorn, 268.8 μg/egg vs. 238.7 μg/egg) (*p* > 0.05). 

It is worth mentioning that our study’s results should be compared to prior research with care. Although there were no notable contrasts in ALA, EPA, DHA, or total n-3 PUFA levels of eggs from different breeds’ CON groups, we detected a substantial difference in the eggs from the FSO groups (*p* < 0.05). To be specific, our study observed that in the FSO groups, the levels of ALA, DHA, and n-3 PUFA in the eggs of Dwarf Layer were significantly amplified compared to the FSO groups for White Leghorn and other three breeds (*p* < 0.05). Conversely, the EPA levels in the eggs of the FSO group for the Dwarf Layer were significantly lower than those in the FSO group of the White Leghorn breed (*p* < 0.05). There are likely two reasons for this difference. First, the White Leghorn may be more efficient at converting ALA to EPA than the Dwarf Layer. Second, the Dwarf Layer may convert more EPA to DHA for deposition in eggs. This may be related to the breed’s lipid metabolism, which has been linked to sex-linked dwarfism in chickens caused by mutations in the growth hormone receptor gene on the Z chromosome [47]. Additionally, Dwarf Layers have been found to have the breed-specific characteristics of conjugated linoleic acid isomers. As such, their yolk lipids tend to be more enriched in fatty acids in response to dietary conjugated linoleic acid than those of White Leghorn hens [48]. Finally, sex-linked dwarf chickens have been found to have a greater deposition of abdominal fat and larger adipocytes than normal Xinghua chickens [47], which may also have an effect on the lipid metabolism and fatty acid composition of their eggs.

Chickens are unable to synthesize ALA and other n-3 PUFA on their own [49,50]. However, it should be noted that by inserting a double bond at the 3rd and 6th carbon positions (counted from the CH_3_ end location), hens are capable of adding further double bonds [49]. The yolk lipids are produced in the liver of the hen and then transported to the yolk using triacylglycerol-enriched very low-density lipoprotein (VLDL) and phospholipid-rich very high-density lipoprotein vitellogenin via serum [51]. In laying hens, a distinct form of VLDL that is exclusively present in the yolk, VLDLy, exists, which is almost half the size of ordinary VLDL. VLDLy associates with apolipoprotein B100 and apovitellenin-1, preventing the action of lipoprotein lipase and allowing for triglyceride deposition in the oocyte in their intact form [52]. Dietary fatty acid composition has a greater influence on the yolk lipid precursor’s fatty acid composition in Dwarf Layer hens than in regular hens. It is plausible that the dwarfing gene could reduce the hepatic synthesis of de novo fatty acids or that dwarf hens may assimilate more dietary lipids into the yolk relative to normal hens [53]. Efficient nutrient absorption by laying hens depends on the condition of the intestinal absorption surface, which is regulated by the morphology of the intestine, specifically the length and recess depth of the intestinal villus. The villi in the small intestine are responsible for nutrient absorption, while the crypts are responsible for the regeneration of villous mucosal cells [54,55]. Moreover, the macroscopic and microstructural integrity of the intestine plays a crucial role in nutrient absorption and growth efficiency of laying hens [56]. An increased nutrient absorption ability in efficient birds can be attributed to their larger duodenal surface area and a greater ratio of villus height to crypt depth than those of non-efficient birds [33]. The difference in n-3 PUFA deposition between breeds may be related to the conversion of fatty acids in the liver and intestinal absorption. Although differences in n-3 PUFA deposition among breeds were compared in this experiment, the reasons for this difference still need further research.

To better understand the mechanisms for the greater deposition of n-3 PUFA in eggs from the Dwarf Layer breed, further research is required. Future studies should aim to investigate the deposition pattern of n-3 PUFA from the perspective of intestinal absorption, liver lipid synthesis, and liver uptake of dietary lipids. Additionally, candidate genes involved in the efficiency of n-3 PUFA deposition should be characterized, including their mutations. This will aid in the development of specialized strains to improve the production efficiency of n-3 PUFA-enriched eggs.

## 5. Conclusions

We found that the egg of Dwarf Layer contains more ALA, DHA, and n-3 PUFA than four other breeds when the diet is supplemented with FSO. The content of selenium and lutein in eggs did not differ significantly between breeds. Therefore, this experiment provides a reference for the selection of different breeds in the production of functional nutrition eggs.

## Figures and Tables

**Table 1:** Ingredients and nutrient composition of the basic diets (%, air-dry basis).

Items	CON	FSO	MFE	SEY
Ingredients				
Corn	59.24	58.99	59.04	59.23
Soybean meal	25.68	24.86	25.55	25.67
Wheat bran	-	2.25	-	-
Palm oil	3.68	-	3.81	3.68
Flaxseed oil	-	2.50	-	-
Selenium yeast	-	-	-	0.02
Marigold flower extract	-	-	0.20	-
Limestone	9.10	9.10	9.10	9.10
CaHPO_4_	1.00	1.00	1.00	1.00
NaCl	0.30	0.30	0.30	0.30
Premix ^1^	1.00	1.00	1.00	1.00
Total	100	100	100	100
Calculated nutrient composition ^2^				
Metabolizable energy, MJ/kg	11.49	11.49	11.49	11.49
Dry matter	86.00	86.00	86.00	86.00
Crude protein	16.50	16.50	16.50	16.50
Calcium	3.60	3.60	3.60	3.60
Total phosphorus	0.60	0.60	0.60	0.60
Lysine	0.83	0.83	0.83	0.83
Methionine	0.36	0.36	0.36	0.36
Methionine + Cysteine	0.65	0.65	0.65	0.65

^1^ Premix provided the following for per kilogram of diet: Vitamin A, 12,500 IU; Vitamin D3, 4125 IU; Vitamin E, 15 IU; Vitamin K, 2 mg; Vitamin B1, 0.98 mg; Vitamin B2, 8.5 mg; Calcium pantothenate, 50 mg; Niacin, 32.5 mg; Pyridoxine, 8 mg; Biotin, 2 mg; Folic acid, 5 mg; Vitamin B12, 5 mg; Cu, 8 mg; I, 1 mg; Fe, 60 mg; Se, 0.3 mg; Mn, 65 mg; Zn, 66 mg; Choline, 1000 mg; Phytase, 1500 FYT; ^2^ Calculated values.

**Table 2:** Analyzed fatty acid composition (% of total fatty acid) of the CON and FSO groups.

Items	Groups	
	CON	FSO
Palmitic acid (C16:0)	14.69	12.58
Stearic acid (C18:0)	2.31	3.09
Oleic acid (C18:1n-9c)	22.89	22.27
Linoleic acid (C18:2n-6c)	55.17	48.52
α-linolenic acid (C18:3n-3)	2.87	11.65

CON = basic diet; FSO = basic diet + 2.5% flaxseed oil + 0.016% Vitamin E. Fatty acids with less than 1% content were not shown on the table.

**Table 3:** Effect of FSO on performance parameters in different breeds (Trial 1).

Items	Groups	Dwarf Layer	White Leghorn	Silky Fowl	Beijing-You Chicken	Shouguang Chicken
Average daily feed intake, g	CON	110.67 ± 0.92 *^b^	88.01 ± 0.25 ^d^	89.98 ± 3.91 ^d^	100.27 ± 5.64 ^c^	131.33 ± 8.25 ^a^
	FSO	104.06 ± 3.89 ^bc^	111.79 ± 4.10 *^b^	87.56 ± 6.79 ^d^	96.59 ± 4.63 ^c^	127.14 ± 8.47 ^a^
Egg weight, g/egg	CON	54.73 ± 0.31 ^b^	56.63 ± 0.65 ^a^	44.25 ± 0.44 ^d^	47.58 ± 0.54 ^c^	47.90 ± 1.14 ^c^
	FSO	55.98 ± 0.71 *^a^	56.53 ± 0.80 ^a^	43.55 ± 1.04 ^c^	47.93 ± 0.40 ^b^	49.00 ± 1.45 ^b^
Feed-to-egg ratio, g: g	CON	2.97 ± 0.03 ^d^	2.94 ± 0.05 ^d^	4.60 ± 0.21 *^a^	3.32 ± 0.29 ^c^	4.11 ± 0.30 ^b^
	FSO	2.80 ± 0.16 ^c^	2.95 ± 0.04 ^bc^	3.81 ± 0.25 ^a^	3.19 ± 0.31 ^b^	3.88 ± 0.32 ^a^
Egg mass, g/d/bird	CON	37.25 ± 0.63 ^a^	29.98 ± 0.62 ^c^	19.61 ± 1.48 ^d^	30.24 ± 1.15 ^c^	32.00 ± 0.55 ^b^
	FSO	37.27 ± 1.23 ^a^	37.94 ± 1.28 *^a^	23.04 ± 1.60 *^d^	30.38 ± 1.66 ^c^	32.84 ± 0.82 ^b^
Egg production, %	CON	68.05 ± 1.47 ^a^	52.98 ± 1.23 ^c^	44.25 ± 2.95 ^d^	63.53 ± 1.76 ^b^	66.80 ± 1.44 ^a^
	FSO	66.58 ± 1.95 ^ab^	67.12 ± 1.73 *^a^	52.83 ± 2.47 *^c^	63.35 ± 2.99 ^b^	67.05 ± 1.14 ^a^

* In the same column and the same breed, means with different superscripts indicate significant differences (*p* < 0.05). ^a–d^ In the same row, means with different superscripts indicate significant differences (*p* < 0.05). Data are expressed as mean ± SD. Values are the mean ± standard deviation (SD) of 75 replicate hens per treatment for the 4-week experimental period. CON = basic diet; FSO = basic diet + 2.5% flaxseed oil + 0.016% Vitamin E.

**Table 4:** Effect of FSO on egg quality parameters in different breeds (Trial 1).

Items	Groups	Dwarf Layer	White Leghorn	Silky Fowl	Beijing-You Chicken	Shouguang Chicken
EST, μm	CON	323.38 ± 27.49 *^bc^	330.39 ± 24.41 ^b^	313.11 ± 31.43 ^c^	347.39 ± 29.04 ^a^	324.03 ± 26.17 ^bc^
	FSO	310.31 ± 29.45 ^b^	345.77 ± 27.87 *^a^	315.94 ± 32.85 ^b^	350.22 ± 23.73 ^a^	320.88 ± 24.97 ^b^
ESS, kg/cm^2^	CON	2.95 ± 0.76 ^b^	2.94 ± 0.70 ^b^	3.51 ± 0.78 ^a^	3.74 ± 1.01 ^a^	3.82 ± 0.85 ^a^
	FSO	2.76 ± 0.49 ^c^	3.17 ± 0.66 ^b^	3.53 ± 0.71 ^a^	3.82 ± 0.78 ^a^	3.82 ± 1.01 ^a^
YC	CON	8.69 ± 0.45 ^b^	8.93 ± 0.58 ^b^	8.29 ± 0.56 ^c^	9.60 ± 0.54 ^a^	9.59 ± 0.59 ^a^
	FSO	8.64 ± 0.47 ^c^	9.00 ± 0.53 ^b^	8.11 ± 0.52 ^d^	9.54 ± 0.61 ^a^	9.47 ± 0.57 ^a^
EW, g	CON	55.46 ± 4.42 ^a^	57.05 ± 3.67 ^a^	42.95 ± 3.64 ^c^	48.37 ± 2.99 ^b^	48.59 ± 3.71 ^b^
	FSO	56.93 ± 3.69 ^a^	57.07 ± 3.64 ^a^	43.20 ± 3.88 ^c^	49.71 ± 4.14 ^b^	50.43 ± 2.42 *^b^
EYW, g	CON	16.10 ± 1.31 ^b^	17.17 ± 1.21 ^a^	14.13 ± 1.51 ^c^	14.08 ± 0.73 ^c^	15.61 ± 0.96 ^b^
	FSO	16.76 ± 1.16 *^a^	16.94 ± 0.97 ^a^	14.76 ± 1.29 ^c^	14.42 ± 1.09 ^c^	15.83 ± 1.17 ^b^
AW, g	CON	34.10 ± 3.56 ^a^	34.34 ± 2.99 ^a^	24.51 ± 2.77 ^c^	28.88 ± 2.07 ^b^	27.94 ± 2.89 ^b^
	FSO	34.93 ± 2.80 ^a^	34.18 ± 2.61 ^a^	24.07 ± 2.91 ^c^	29.93 ± 2.94 ^b^	29.29 ± 2.00 *^b^
AH, mm	CON	6.81 ± 0.92 ^a^	5.60 ± 0.75 ^bc^	5.16 ± 1.14 ^c^	5.82 ± 1.08 ^b^	5.42 ± 0.96 ^bc^
	FSO	6.84 ± 0.76 ^a^	5.63 ± 1.00 ^b^	4.88 ± 0.84 ^c^	5.74 ± 0.72 ^b^	5.66 ± 1.00 ^b^
HU	CON	83.82 ± 5.28 ^a^	74.30 ± 5.76 ^c^	77.22 ± 7.57 ^bc^	78.90 ± 7.48 ^b^	76.06 ± 7.31 ^bc^
	FSO	83.18 ± 4.79 ^a^	74.25 ± 7.64 ^c^	74.32 ± 5.92 ^c^	78.24 ± 5.01 ^b^	77.23 ± 6.94 ^bc^

* In the same column and the same breed, means with different superscripts indicate significant differences (*p* < 0.05). ^a–c^ In the same row, means with different superscripts indicate significant differences (*p* < 0.05). Data are expressed as mean ± SD. Values are the mean ± standard deviation (SD) of 30 replicate hens per treatment for the 4-week experimental period. EST = eggshell thickness; ESS = eggshell strength; YC = yolk color; EW = egg weight; EYW = egg yolk weight; AW = albumen weight; AH = albumen height; HU = Haugh units; CON = a basic diet; FSO = basic diet + 2.5% flaxseed oil + 0.016% Vitamin E.

**Table 5:** Effect of functional dietary nutrients on the performance parameters in different breeds (Trial 2).

Items	Groups	Dwarf Layer	White Leghorn
Average daily feed intake, g	CON	104.89 ± 1.80	108.51 ± 5.34 ^b^
	FSO	94.30 ± 8.67	107.29 ± 3.09 ^b^
	SEY	113.08 ± 5.20	116.13 ± 2.45 ^a^
	MFE	99.75 ± 15.95	110.03 ± 1.95 ^ab^
Egg weight, g/egg	CON	50.19 ± 0.42 *^a^	47.83 ± 0.50
	FSO	48.99 ± 0.57 ^b^	48.92 ± 0.48
	SEY	50.45 ± 0.82 ^a^	48.76 ± 0.74
	MFE	50.71 ± 0.48 *^a^	48.16 ± 0.84
Feed-to-egg ratio, g: g	CON	2.63 ± 0.11	2.97 ± 0.56
	FSO	2.49 ± 0.27	2.68 ± 0.37
	SEY	2.53 ± 0.06	2.88 ± 0.25
	MFE	2.26 ± 0.36	2.94 ± 0.29
Egg mass, g/d/bird	CON	39.93 ± 1.98 ^b^	37.20 ± 5.87
	FSO	37.90 ± 1.74 ^b^	40.49 ± 4.34
	SEY	44.63 ± 1.74 ^a^	40.56 ± 3.55
	MFE	44.17 ± 0.07 ^a^	37.72 ± 4.19
Egg production, %	CON	79.55 ± 3.85 ^b^	77.77 ± 12.02
	FSO	77.37 ± 3.11 ^b^	82.72 ± 8.10
	SEY	88.44 ± 2.77 ^a^	83.11 ± 6.01
	MFE	87.11 ± 0.77 ^a^	78.22 ± 7.34

* In the same row, means with different superscripts indicate significant differences (*p* < 0.05). ^a,b^ In the same column and the same breed, means with different superscripts indicate significant differences (*p* < 0.05). Data are expressed as mean ± SD. Values are the mean ± standard deviation (SD) of 75 replicate hens per treatment for the 4-week experimental period. CON = a basic diet; FSO = basic diet + 2.5% flaxseed oil + 0.016% Vitamin E; SEY = CON + 0.02% selenium yeast; MFE = CON + 0.2% marigold flower extract.

**Table 6:** Effect of functional dietary nutrients on egg quality parameters in different breeds (Trial 2).

Items	Groups	Dwarf Layer	White Leghorn
ESS, kg/cm^2^	CON	3.22 ± 0.75	3.48 ± 0.85
	FSO	3.06 ± 0.74	3.58 ± 0.77 *
	SEY	3.18 ± 0.54	3.54 ± 0.85
	MFE	3.16 ± 0.74	3.58 ± 0.64 *
YC	CON	8.26 ± 0.45 ^bc^	8.23 ± 0.67 ^b^
	FSO	8.19 ± 0.64 ^c^	8.44 ± 0.70 ^b^
	SEY	8.60 ± 0.67 ^b^	8.28 ± 0.60 ^b^
	MFE	9.26 ± 0.69 ^a^	9.06 ± 0.37 ^a^
EW, g	CON	49.40 ± 4.51 ^b^	47.89 ± 3.47
	FSO	49.15 ± 4.25 ^b^	49.29 ± 3.46
	SEY	51.71 ± 4.69 *^a^	48.10 ± 3.16
	MFE	50.52 ± 3.53 *^ab^	47.62 ± 2.79
EYW, g	CON	13.19 ± 1.38 b	13.15 ± 1.08
	FSO	13.15 ± 1.78 b	13.40 ± 1.02
	SEY	14.11 ± 1.17 *a	13.04 ± 0.62
	MFE	13.77 ± 1.38 *ab	13.08 ± 0.91
AW, g	CON	31.42 ± 3.44 *	29.63 ± 2.52
	FSO	31.25 ± 2.87	30.60 ±2.58
	SEY	32.38 ± 3.64 *	29.77 ± 2.56
	MFE	31.86 ± 2.41 *	29.33 ± 2.12
AH, mm	CON	7.11 ± 0.82 *	6.67 ± 0.84
	FSO	7.50 ± 0.86 *	6.34 ± 0.71
	SEY	7.57 ± 1.04 *	6.54 ± 0.75
	MFE	7.34 ± 1.00 *	6.42 ± 0.97
HU	CON	87.97± 4.65 *	85.20 ± 4.98
	FSO	89.59 ± 4.51 *	82.87 ± 4.49
	SEY	89.18 ± 5.77 *	84.38 ± 4.36
	MFE	88.28 ± 5.54 *	83.59 ± 6.20

* In the same row, means with different superscripts indicate significant differences (*p* < 0.05). ^a–c^ In the same column and the same breed, means with different superscripts indicate significant differences (*p* < 0.05). Data are expressed as mean ± SD. Values are the mean ± standard deviation (SD) of 30 replicate hens per treatment for the 4-week experimental period. ESS = eggshell strength; YC = yolk color; EW = egg weight; EYW = egg yolk weight; AW = albumen weight; AH = albumen height; HU = Haugh units; CON = a basic diet; FSO = basic diet + 2.5% flaxseed oil + 0.016% Vitamin E; SEY = CON + 0.02% selenium yeast; MFE = CON + 0.2% marigold flower extract.

## Data Availability

The data that support the findings of this study are available upon request to the corresponding author. The data are not publicly available due to privacy or ethical restrictions.

## Supplementary Materials

The following supporting information can be downloaded at https://www.mdpi.com/article/10.3390/ani13193066/s1, Table S1: Fatty acids composition (%) of eggs from different breeds with FSO dietary in Trial 1; Table S2: Fatty acids composition of eggs from different breeds with FSO dietary in Trial 1; Table S3: Fatty acids composition (%) of eggs from different breeds with FSO dietary in Trial 2; Table S4: Fatty acids composition of eggs from different breeds with FSO dietary in Trial 2.
