# Supplementary material for: Effects of Breeds on the Content of Functional Nutrition in Eggs
Source: Animals (Basel). 2023 Sep 29;13(19):3066. doi: 10.3390/ani13193066 (PMC10571526; doi:10.3390/ani13193066)
Supplement: Supplementary file 1 [file animals-13-03066-s001.zip › animals-2572976-supplementary.pdf]

Table S1. Fatty acids composition (%) of eggs from different breeds with FSO in Trial 1.

Items	Dwarf Layer		White Leghorn		Silky fowl		Beijing-you chicken		Shouguang chicken		SEM	*P* value		
	CON	FSO	CON	FSO	CON	FSO	CON	FSO	CON	FSO		Breed	Diet	Breed* Diet
C12:0	0.01^{a}	0.01^{abc}	0.01^{ab}	0.01^{ab}	0.01^{abc}	0.01^{abc}	0.01^{abc}	0.01^{c}	0.01^{abc}	0.01^{bc}	0.00	0.07	0.04	0.28
C14:0	0.36^{a}	0.27^{cd}	0.33^{a}	0.25^{d}	0.31^{abc}	0.25^{d}	0.34^{a}	0.28^{bcd}	0.36^{a}	0.33^{ab}	0.02	0.03	< 0.01	0.38
C15:0	0.06 ^{a}	0.06^{ab}	0.06^{ab}	0.05^{ab}	0.06^{ab}	0.05^{b}	0.05^{ab}	0.06^{ab}	0.05^{ab}	0.06^{ab}	0.00	0.38	0.26	0.12
C16:0	26.49^{a}	22.89^{c}	25.99^{a}	23.81^{bc}	26.22^{a}	23.82^{bc}	25.67^{a}	22.91^{c}	26.81^{a}	25.07^{ab}	0.57	0.09	< 0.01	0.57
C17:0	0.17^{b}	0.18^{b}	0.18^{b}	0.17^{b}	0.18^{b}	0.18^{b}	0.19^{ab}	0.21^{a}	0.16^{b}	0.18^{b}	0.01	0.02	0.08	0.47
C18:0	9.37	8.91	9.58	9.93	9.42	9.75	9.63	10.05	9.96	9.41	0.34	0.3	0.93	0.43
C20:0	0.14^{b}	0.37^{a}	0.19^{ab}	0.38^{a}	0.09^{b}	0.36^{a}	0.05^{b}	0.05^{b}	0.06^{b}	0.06^{b}	0.07	< 0.01	< 0.01	0.15
C21:0	0.29^{a}	0.21^{bcd}	0.29^{a}	0.17^{d}	0.27^{a}	0.15^{d}	0.24^{abc}	0.21^{bcd}	0.26^{ab}	0.18^{cd}	0.02	0.28	< 0.01	0.13
C22:0	0.04	0.03	0.04	0.03	0.04	0.03	0.04	0.04	0.04	0.04	0.01	0.77	0.05	0.68
C23:0	0.03^{a}	0.00^{b}	0.03^{ab}	0.00^{b}	0.03^{a}	0.00^{b}	0.04^{a}	0.00^{b}	0.04^{a}	0.00^{b}	0.01	0.92	< 0.01	0.92
C24:0	0.13^{ab}	0.03^{c}	0.13^{ab}	0.03^{c}	0.13^{ab}	0.03^{c}	0.21^{a}	0.19^{a}	0.19^{a}	0.18^{a}	0.03	< 0.01	0.01	0.47
C14:1	0.07^{ab}	0.04^{bcd}	0.06^{abcd}	0.04^{cd}	0.05^{abcd}	0.03^{d}	0.06^{abc}	0.04^{cd}	0.07^{a}	0.06^{abcd}	0.01	0.06	< 0.01	0.97
C16:1	2.84^{a}	2.23^{abc}	2.52^{ab}	2.12^{abc}	2.51^{ab}	1.90^{bc}	2.26^{abc}	1.77^{c}	2.75^{a}	2.44^{abc}	0.22	0.09	< 0.01	0.95
C18:1n9c	37.85^{ab}	38.45^{ab}	37.94^{ab}	39.43^{a}	38.16^{ab}	39.36^{a}	38.73^{ab}	37.72^{ab}	38.97^{ab}	37.15^{b}	0.63	0.72	0.82	0.06
C20:1	0.26^{a}	0.23^{ab}	0.25^{a}	0.22 ^{ab}	0.23^{ab}	0.19^{b}	0.22^{ab}	0.21^{ab}	0.25^{a}	0.20^{ab}	0.02	0.34	0.01	0.79
C22:1n9	0.03^{a}	0.00^{b}	0.03^{a}	0.00^{b}	0.02^{ab}	0.00^{b}	0.03^{a}	0.02^{ab}	0.03^{a}	0.03^{a}	0.01	0.08	< 0.01	0.43
C24:1	0.05	0.04	0.05	0.05	0.04	0.05	0.06	0.04	0.06	0.06	0.00	0.14	0.72	0.37
C20:2	0.03	0.04	0.02	0.04	0.02	0.03	0.00	0.03	0.00	0.03	0.01	0.51	0.04	0.89
C22:2	0.02^{b}	0.06^{a}	0.04^{ab}	0.04^{ab}	0.03^{ab}	0.03^{ab}	0.04^{ab}	0.05^{ab}	0.04^{ab}	0.03^{ab}	0.01	0.6	0.2	0.24
C18:2n6c	17.86^{ab}	17.77^{abc}	18.43^{ab}	16.55^{bc}	18.41^{ab}	16.9^{abc}	17.91^{ab}	18.79^{a}	15.96^{c}	17.35^{abc}	0.56	0.09	0.51	0.03
C20:3n6	0.24^{a}	0.17^{b}	0.21^{ab}	0.19^{ab}	0.22^{ab}	0.17^{b}	0.20^{ab}	0.19^{ab}	0.21^{ab}	0.17^{b}	0.02	0.92	< 0.01	0.55
C20:4n6	2.38^{b}	1.55^{d}	2.53^{ab}	1.55^{d}	2.49^{ab}	1.68^{d}	2.77^{a}	2.00^{c}	2.58^{ab}	1.67^{d}	0.09	< 0.01	< 0.01	0.82

C18:3n3	0.63^{c}	4.28^{a}	0.41^{c}	3.29^{b}	0.40^{c}	3.11^{b}	0.43^{c}	3.12^{b}	0.39^{c}	3.46^{b}	0.13	< 0.01	< 0.01	0.01
C20:3n3	0.03^{c}	0.09^{a}	0.03^{c}	0.06^{b}	0.03^{c}	0.06^{b}	0.03^{c}	0.07^{ab}	0.03^{c}	0.06^{b}	0.01	0.25	< 0.01	0.28
C20:5n3	0.01^{c}	0.07^{c}	0.01^{c}	0.09^{a}	0.01^{c}	0.09^{ab}	0.00^{c}	0.09^{ab}	0.01^{c}	0.10^{a}	0.01	0.25	< 0.01	0.29
C22:6n3	0.62^{d}	2.03^{a}	0.67^{d}	1.49^{c}	0.63^{d}	1.76^{b}	0.79^{d}	1.86^{ab}	0.72^{d}	1.71^{b}	0.06	< 0.01	< 0.01	< 0.01
∑SFA	37.10^{ab}	32.96^{e}	36.80^{ab}	34.83^{cd}	36.75^{ab}	34.63^{de}	36.46^{abc}	34.00^{de}	37.92^{a}	35.50^{bcd}	0.55	0.05	< 0.01	0.31
∑MUFA	41.09^{ab}	41.00^{ab}	40.84^{ab}	41.85^{a}	41.02^{ab}	41.53^{ab}	41.36^{ab}	39.80^{b}	42.14^{a}	39.93^{b}	0.54	0.66	0.19	0.04
∑PUFA	21.81^{c}	26.05^{a}	22.35^{c}	23.31^{bc}	22.23^{c}	23.84^{bc}	22.18^{c}	26.20^{a}	19.94^{d}	24.57^{ab}	0.62	0.03	< 0.01	0.02
∑n6	20.47^{abc}	19.49^{abcd}	21.18^{a}	18.29^{d}	21.12^{a}	18.75^{cd}	20.8^{ab}	20.98^{ab}	18.75^{cd}	19.19^{bcd}	0.58	0.05	0.01	0.03
∑n-3	1.29^{c}	6.46^{a}	1.12^{c}	4.94^{b}	1.06 ^{c}	5.02^{b}	1.26^{c}	5.14^{b}	1.15^{c}	5.33^{b}	0.14	< 0.01	< 0.01	< 0.01
n6/n3	16.69^{b}	3.02^{c}	19.34^{ab}	3.71^{c}	19.99^{a}	3.73^{c}	16.63^{b}	4.09^{c}	16.35^{b}	3.60^{c}	1.01	0.20	< 0.01	0.27

$^{a-e}$In the same row, means with different superscripts indicate significant differences (*P* < 0.05).

SFA = saturated acid; MUFA = mono unsaturated fatty acid; PUFA = polyunsaturated fatty acid; ∑= Total; ∑n-3 = 18:3n3 + 20:5n3 + 22:6n3; ∑n-6 = 18:2n6 + 20:3n6 + 20:4n6; n-6/n-3 = ∑n-6/∑n-3; CON = a control diet; FSO = CON + 2.5% flaxseed oil + 0.016% Vitamin E.

Table S2. Fatty acids composition of eggs from different breeds with FSO in Trial 1.

Items	Items	Dwarf Layer		White Leghorn		Silky fowl		Beijing-you chicken		Shouguang chicken		SEM	*P* value		
		CON	FSO	CON	FSO	CON	FSO	CON	FSO	CON	FSO		Breed	Diet	Breed* Diet
mg/egg	ALA	30.55^{d}	212.45^{a}	21.21^{d}	162.05^{bc}	17.96^{d}	136.13^{c}	20.48^{d}	134.73^{c}	19.93^{d}	172.79^{b}	9.57	< 0.01	< 0.01	0.01
	EPA	0.32^{c}	3.39^{b}	0.41^{c}	4.42^{ab}	0.49^{c}	3.78^{b}	0.31^{c}	3.71^{b}	0.37^{c}	4.79^{a}	0.30	0.13	< 0.01	0.17
	DHA	29.29^{d}	100.32^{a}	35.18^{d}	73.13^{c}	28.37^{d}	77.05^{bc}	38.18^{d}	80.47^{bc}	37.07^{d}	85.49^{b}	3.24	< 0.01	< 0.01	< 0.01
	∑n3	61.58^{d}	320.59^{a}	58.32^{d}	242.71^{bc}	48.06^{d}	219.73^{c}	60.37^{d}	222.16^{c}	59.08^{d}	266.14^{b}	11.10	< 0.01	< 0.01	< 0.01
mg/g yolk	ALA	1.91^{c}	12.93^{a}	1.23^{c}	10.04^{b}	1.25^{c}	9.97^{b}	1.44^{c}	9.73^{b}	1.25^{c}	11.12^{b}	0.59	0.03	< 0.01	0.18
	EPA	0.02^{c}	0.21^{b}	0.02^{c}	0.27^{a}	0.03^{c}	0.28^{a}	0.02^{c}	0.27^{a}	0.02^{c}	0.31^{a}	0.02	0.11	< 0.01	0.18
	DHA	1.84^{d}	6.10^{a}	2.04^{d}	4.53^{b}	1.98^{d}	5.64^{a}	2.68^{d}	5.81^{a}	2.33^{d}	5.50^{a}	0.21	< 0.01	< 0.01	< 0.01
	∑n3	3.86^{c}	19.51^{a}	3.38^{c}	15.04^{b}	3.36^{c}	16.09^{b}	4.23^{c}	16.04^{b}	3.72^{c}	17.13^{b}	0.69	0.02	< 0.01	0.06
mg/g egg	ALA	0.55^{d}	3.79^{a}	0.37^{d}	2.95^{bc}	0.40^{d}	3.29^{ab}	0.41^{d}	2.74^{ab}	0.41^{d}	3.43^{ab}	0.17	0.02	< 0.01	0.11
	EPA	0.01^{d}	0.06^{c}	0.01^{d}	0.08^{ab}	0.01^{d}	0.09^{ab}	0.01^{d}	0.08^{ab}	0.01^{d}	0.10^{a}	0.01	0.02	< 0.01	0.08
	DHA	0.53^{e}	1.79^{ab}	0.61^{e}	1.33^{c}	0.63^{e}	1.86^{ab}	0.77^{e}	1.64^{ab}	0.75^{e}	1.70^{ab}	0.06	< 0.01	< 0.01	< 0.01
	∑n3	1.11^{c}	5.72^{a}	1.01^{c}	4.42^{b}	1.07^{c}	5.31^{a}	1.22^{c}	4.52^{a}	1.20^{c}	5.29^{a}	0.20	0.02	< 0.01	0.02
mg/100g egg	ALA	54.99^{d}	378.83^{a}	36.77^{d}	295.32^{bc}	40.08^{d}	328.86^{ab}	41.42^{d}	273.96^{c}	40.51^{d}	343.39^{ab}	17.34	0.02	< 0.01	0.11
	EPA	0.57^{d}	6.05^{c}	0.72^{d}	8.05^{ab}	1.10^{d}	9.13^{ab}	0.63^{d}	7.54^{bc}	0.76^{d}	9.51^{a}	0.55	0.02	< 0.01	0.08
	DHA	52.73^{e}	178.89^{ab}	61.00^{de}	133.26^{c}	63.32^{de}	186.13^{a}	77.21^{d}	163.63^{b}	75.34^{d}	169.89^{ab}	6.25	< 0.01	< 0.01	< 0.01
	∑n-3	110.85^{c}	571.66^{a}	101.13^{c}	442.31^{b}	107.27^{c}	530.84^{a}	122.07^{c}	451.73^{b}	120.07^{c}	528.89^{a}	20.09	0.02	< 0.01	0.02

$^{a-e}$ In the same row, means with different superscripts indicate significant differences ($P < 0.05$).

ALA = α-linolenic acid (C18:3); EPA = eicosapentaenoic acid (C20:5); DHA = docosahexaenoic acid (C22:6); ∑n-3 = ALA + EPA + DHA; CON = a control diet; FSO = CON + 2.5% flaxseed oil + 0.016% Vitamin E.

Table S3. Fatty acids composition (%) of eggs from different breeds with FSO dietary in Trial 2.

Items	Dwarf Layer		White Leghorn		*SEM*	*P* value		
	CON	FSO	CON	FSO		Breed	Diet	Breed* Diet
C10:0	0.01[a]	0.01[a]	0.01[a]	0.01[b]	0.00	0.35	0.03	< 0.01
C12:0	0.01[b]	0.01[b]	0.01[a]	0.01[b]	0.00	0.05	< 0.01	0.12
C14:0	0.31[ab]	0.23[c]	0.31[a]	0.28[b]	0.01	0.01	< 0.01	0.02
C15:0	0.05	0.06	0.05	0.05	0.01	0.19	0.81	0.29
C16:0	25.45[a]	22.18[c]	26.02[a]	24.30[b]	0.46	< 0.01	< 0.01	0.02
C17:0	0.18[b]	0.21[a]	0.19[ab]	0.17[b]	0.01	0.10	0.63	0.01
C18:0	9.10[b]	9.04[b]	9.77[a]	9.45[ab]	0.29	0.01	0.36	0.52
C20:0	0.06[a]	0.05[b]	0.05[b]	0.05[b]	0.00	0.07	< 0.01	0.01
C21:0	0.32[a]	0.21[c]	0.27[b]	0.19[c]	0.02	0.01	< 0.01	0.34
C22:0	0.03[b]	0.03[b]	0.03[b]	0.04[a]	0.00	0.1	< 0.01	0.20
C23:0	0.01[b]	0.02[a]	0.02[b]	0.02[a]	0.00	0.13	< 0.01	0.02
C24:0	0.02	0.02	0.02	0.01	0.00	0.81	0.24	0.37
C14:1	0.06[a]	0.03[b]	0.05[a]	0.05[a]	0.01	0.33	0.02	0.02
C16:1	2.58[a]	1.88[b]	2.41[a]	2.62[a]	0.22	0.08	0.13	0.01
C18:1n9c	40.61[a]	37.93[ab]	37.65[ab]	36.75[b]	1.39	0.04	0.08	0.37
C20:1	0.29[a]	0.19[c]	0.22[b]	0.17[c]	0.01	< 0.01	< 0.01	0.02

C22:1n9	0.01	0.01	0.01	0.01	0.00	0.28	0.54	0.90
C24:1	0.04	0.03	0.04	0.03	0.00	0.11	0.01	0.73
C20:2	0.01	0.01	0.01	0.01	0.00	0.74	0.56	0.37
C18:2n6c	17.29	19.30	19.23	18.69	1.09	0.40	0.35	0.11
C20:3n6	0.19^{a}	0.16^{b}	0.19^{a}	0.18^{ab}	0.01	0.22	0.05	0.32
C20:4n6	2.35^{a}	1.62^{b}	2.43^{a}	1.65^{b}	0.07	0.29	< 0.01	0.60
C20:3n3	0.03^{c}	0.09^{a}	0.01^{d}	0.07^{b}	0.01	< 0.01	< 0.01	0.96
C18:3n3	0.37^{c}	4.54^{a}	0.36^{c}	3.52^{b}	0.30	0.02	< 0.01	0.02
C20:5n3	0.01^{c}	0.08^{b}	0.01^{c}	0.09^{a}	0.01	0.12	< 0.01	0.17
C22:6n3	0.61^{c}	2.05^{a}	0.63^{c}	1.59^{b}	0.07	< 0.01	< 0.01	< 0.01
∑SFA	35.55^{b}	32.06^{d}	36.76^{a}	34.58^{c}	0.38	< 0.01	< 0.01	0.02
∑MUFA	43.58^{a}	40.08^{b}	40.38^{b}	39.63^{b}	1.36	0.07	0.03	0.16
∑PUFA	20.86^{b}	27.85^{a}	22.86^{b}	25.79^{a}	1.27	0.97	< 0.01	0.03
∑n6	19.83	21.08	21.84	20.51	1.08	0.35	0.96	0.10
∑n3	0.99^{c}	6.67^{a}	1.00^{c}	5.20^{b}	0.32	< 0.01	< 0.01	< 0.01
n6: n3	19.34^{b}	3.18^{c}	21.58^{a}	3.93^{c}	0.55	< 0.01	< 0.01	0.07

[a-d] In the same row, means with different superscripts indicate significant differences ($P < 0.05$).

SFA = saturated acid; MUFA = mono unsaturated fatty acid; PUFA = polyunsaturated fatty acid; ∑ = Total; ∑n-3 = 18:3n3 + 20:5n3 + 22:6n3; ∑n-6 = 18:2n6 + 20:3n6 + 20:4n6; n-6/n-3 = ∑n-6 /∑n-3; CON = a control diet; FSO = CON + 2.5% flaxseed oil + 0.016% Vitamin E.

Table S4. Fatty acids composition of eggs from different breeds with FSO dietary in Trial 2.

Items	Groups	Dwarf Layer		White Leghorn		*SEM*	*P* value		
		CON	FSO	CON	FSO		Breed	Diet	Breed* Diet
mg/egg	ALA	14.01[c]	163.15[a]	13.44[c]	139.76[b]	10.98	0.13	< 0.01	0.15
	EPA	0.47[c]	2.96[b]	0.49[c]	3.69[a]	0.19	< 0.01	< 0.01	0.01
	DHA	23.6[c]	74.34[a]	22.89[c]	62.77[b]	2.81	< 0.01	< 0.01	0.01
	∑n3	38.07[c]	240.45[a]	36.82[c]	206.22[b]	11.96	0.04	< 0.01	0.06
mg/g yolk	ALA	1.11[c]	12.13[a]	0.98[c]	10.47[b]	0.82	0.13	< 0.01	0.19
	EPA	0.04[c]	0.22[b]	0.04[c]	0.28[a]	0.01	0.01	< 0.01	< 0.01
	DHA	1.88[c]	5.53[a]	1.67[c]	4.70[b]	0.21	< 0.01	< 0.01	0.04
	∑n3	3.03[c]	17.88[a]	2.69[c]	15.45[b]	0.89	0.04	< 0.01	0.11
mg/g egg	ALA	0.29[b]	3.26[a]	0.28[b]	2.83[a]	0.22	0.17	< 0.01	0.19
	EPA	0.01[c]	0.06[b]	0.01[c]	0.07[a]	0.00	< 0.01	< 0.01	< 0.01
	DHA	0.49[c]	1.48[a]	0.47[c]	1.27[b]	0.06	< 0.01	< 0.01	0.02
	∑n3	0.78[c]	4.80[a]	0.76[c]	4.17[b]	0.24	0.06	< 0.01	0.08
mg/100g egg	ALA	28.80[b]	325.52[a]	27.74[b]	282.64[a]	21.97	0.17	< 0.01	0.19
	EPA	0.96[c]	5.91[b]	1.01[c]	7.46[a]	0.39	< 0.01	< 0.01	< 0.01
	DHA	48.52[c]	148.33[a]	47.24[c]	126.94[b]	5.67	< 0.01	< 0.01	0.02
	∑n-3	78.27[c]	479.75[a]	75.98[c]	417.04[b]	23.96	0.06	< 0.01	0.08

[a-c] In the same row, means with different superscripts indicate significant differences ($P < 0.05$).

ALA = α-linolenic acid (C18:3); EPA = eicosapentaenoic acid (C20:5); DHA = docosahexaenoic acid (C22:6); ∑n-3 = ALA + EPA + DHA; CON = a control diet; FSO = CON + 2.5% flaxseed oil + 0.016% Vitamin.
